# Evidence That Emmetropization Buffers Against Both Genetic and Environmental Risk Factors for Myopia

**DOI:** 10.1167/iovs.61.2.41

**Published:** 2020-02-25

**Authors:** Alfred Pozarickij, Clair A. Enthoven, Neema Ghorbani Mojarrad, Denis Plotnikov, Milly S. Tedja, Annechien E G. Haarman, J. Willem L. Tideman, Jan Roelof Polling, Kate Northstone, Cathy Williams, Caroline C. W. Klaver, Jeremy A. Guggenheim

**Affiliations:** 1 School of Optometry & Vision Sciences, Cardiff University, Cardiff, United Kingdom; 2 Department of Ophthalmology, Erasmus University Medical Center, Rotterdam, The Netherlands; 3 The Generation R Study Group, Erasmus University Medical Center, Rotterdam, The Netherlands; 4 Department of Epidemiology, Erasmus University Medical Center, Rotterdam, The Netherlands; 5 Department of Orthoptics & Optometry, University of Applied Sciences, Faculty of Health, Utrecht, The Netherlands; 6 Population Health Sciences, Bristol Medical School, University of Bristol, Bristol, United Kingdom; 7 Department of Ophthalmology, Donders Institute for Brain, Cognition and Behaviour, Radboud University Medical Center, Nijmegen, The Netherlands; 8 Institute of Molecular and Clinical Ophthalmology, Basel, Switzerland

**Keywords:** refractive error, myopia, ALSPAC, Generation R, genetic epidemiology

## Abstract

**Purpose:**

To test the hypothesis that emmetropization buffers against genetic and environmental risk factors for myopia by investigating whether risk factor effect sizes vary depending on children's position in the refractive error distribution.

**Methods:**

Refractive error was assessed in participants from two birth cohorts: Avon Longitudinal Study of Parents and Children (ALSPAC) (noncycloplegic autorefraction) and Generation R (cycloplegic autorefraction). A genetic risk score for myopia was calculated from genotypes at 146 loci. Time spent reading, time outdoors, and parental myopia were ascertained from parent-completed questionnaires. Risk factors were coded as binary variables (0 = low, 1 = high risk). Associations between refractive error and each risk factor were estimated using either ordinary least squares (OLS) regression or quantile regression.

**Results:**

Quantile regression: effects associated with all risk factors (genetic risk, parental myopia, high time spent reading, low time outdoors) were larger for children in the extremes of the refractive error distribution than for emmetropes and low ametropes in the center of the distribution. For example, the effect associated with having a myopic parent for children in quantile 0.05 vs. 0.50 was as follows: ALSPAC: age 15, –1.19 D (95% CI –1.75 to –0.63) vs. –0.13 D (–0.19 to –0.06), *P* = 0.001; Generation R: age 9, –1.31 D (–1.80 to –0.82) vs. –0.19 D (–0.26 to –0.11), *P* < 0.001. Effect sizes for OLS regression were intermediate to those for quantiles 0.05 and 0.50.

**Conclusions:**

Risk factors for myopia were associated with much larger effects in children in the extremes of the refractive error distribution, providing indirect evidence that emmetropization buffers against both genetic and environmental risk factors.

Myopia is a common eye disorder most often caused by axial elongation of the eye in childhood and adolescence. The prevalence of myopia is rising dramatically; 50% of young adults in Europe and 80% in urban areas in China are currently estimated to be myopic.^1^^,^^2^ Myopia is associated with retinal complications in adulthood, such as myopic macular degeneration, retinal detachment, and glaucoma.^3^^–^^5^ It is currently a leading cause of irreversible visual impairment and blindness.^5^^,^^6^

Experimental models suggest that the development of myopia is a consequence of the emmetropization process influenced by a combination of genetic and environmental factors.^7^ Genome-wide association studies have identified more than 150 genetic variants associated with refractive error.^8^ Together, these genetic variants explain ∼8% of the phenotypic variance in adults and ∼2% in children.^8^^,^^9^ Near work and lack of outdoor exposure are important environmental risk factors associated with myopia.^10^^,^^11^ Recent meta-analyses reported a 85% increased odds of myopia in children who performed a “high” versus “low” level of near work and a 2% increased odds for every one diopter-hour of more near work per week, whereas 4.5 to 7.5 additional hours of outdoor exposure was associated with a 43% reduction in the risk of incident myopia.^10^^,^^11^ Parental myopia is another important risk factor and is often used as a proxy for genetic predisposition but may also involve shared environmental effects.^9^^,^^12^ Many studies have reported an association between sex and myopia,^13^^,^^14^ usually with myopia being more common in girls than boys. This association may be caused in part by the association between puberty and myopia,^15^ coupled with the earlier age of onset of puberty in girls.

Myopia and refractive error have been extensively investigated using conventional ordinary least squares (OLS) linear and logistic regression in order to quantify the effects of genetic and environmental risk factors. In OLS analyses, it is assumed that the “effect size” of each risk factor is consistent across the whole study population.^16^ However, studies on other continuous phenotypes such as body mass index (BMI), height, and birth weight have demonstrated that the effect of genetic and environmental factors can differ for individuals depending on where they lie in the phenotypic distribution.^17^^–^^20^ For example, Williams^19^ reported that a polygenic risk score quantifying a person's genetic predisposition to a high or low BMI was associated with a 4.2-fold larger effect size in obese compared with very lean individuals. In contrast to OLS regression, conditional quantile regression (CQR) can be used to determine the effect associated with a risk factor in specific quantiles of the phenotypic distribution.^16^ In the current study, we applied CQR to test the hypothesis that genetic and environmental risk factors for myopia exert larger effects in some children than others. Specifically, we explored whether the magnitude (in diopters) of risk factor–refractive error associations was larger in children who already had relatively high levels of ametropia.

## Methods

### Avon Longitudinal Study of Parents and Children (ALSPAC)

Pregnant women resident in Avon, England, were recruited between April 1991 and December 1992. Of the initial pregnancies, there were 13,988 children who were alive at 1 year of age. When the oldest children were approximately 7 years of age, an attempt was made to bolster the initial sample with eligible cases who had failed to join the study originally. Accordingly, the total sample size increased to 15,454 pregnancies. Of these, 14,901 were alive at 1 year of age. Information on the cohort parents and their offspring was collected using a variety of methodologies, including self-completion questionnaires sent to study mothers, fathers, teachers, and the study child; direct examination at the research clinic using standardized protocols; and linkage to educational data from the school system.^21^^,^^22^ The ALSPAC study website contains details of all the data that are available through a fully searchable data dictionary and variable search tool: http://www.bristol.ac.uk/alspac/researchers/our-data/.

Ethical approval for the study was obtained from the ALSPAC Ethics and Law Committee (ALEC; IRB00003312; registered as “U Bristol IRB #1” on the Office of Human Research Protections database) and the Local Research Ethics Committees. Informed consent for the use of data collected via questionnaires and clinics was obtained from participants following the recommendations of the ALEC at the time. Detailed information describing how the confidentiality of the cohort is maintained can be found at http://www.bristol.ac.uk/alspac/researchers/research-ethics/.

### The Generation R Study

In this population-based prospective cohort study based in Rotterdam, The Netherlands, pregnant women were recruited between April 2002 and January 2006.^23^^,^^24^ Of the 9778 mothers enrolled in the study, 9749 gave birth to live born children. The exact methodology of the Generation R study has been described elsewhere.^23^^,^^24^ In short, information on the cohort parents and their offspring was collected by direct examination at the research clinic using standardized protocols, magnetic resonance imaging (MRI), urine and blood samples, interviews, and questionnaires. Children were invited to the research center at the age of 9 years, and 5862 (60%) of them participated. The study protocol was approved by the Medical Ethical Committee of the Erasmus Medical Centre, Rotterdam (MEC 217.595/2002/20), and conducted according to the Declaration of Helsinki. Written informed consent was obtained from all participants. More information in the study cohort measurements and collaborations can be found at https://generationr.nl/researchers/.

### Refractive Error

ALSPAC participants were invited to attend a research clinic approximately once per year from the age of 7. For the research clinic visits scheduled when the children were aged 7, 10, 11, 12, and 15 years, noncycloplegic autorefraction was performed using a Canon R50 instrument (Canon USA, Inc., Lake Success, NY, USA). Generation R participants were invited to a research center at the age 9 years. The institutional review board approved the installation of cycloplegic eye drops midway through these research clinic visits; hence, a proportion of the 9-year-old participants received automated cycloplegic refractive error using a Topcon KR8900 instrument (Topcon, Tokyo, Japan). Specifically, 2395 (41.8%) of the 5862 Generation R attendees received cycloplegia, which consisted of two drops (three in case of dark irises) of 1% cyclopentolate instilled at 5-minute intervals at least 30 minutes before autorefraction. Pupil diameter was ≥6 mm at the time of measurement. Spherical equivalent was calculated as the sum of the full spherical value and half of the cylindrical value.

### Questionnaire-Derived Risk Factors

#### ALSPAC

When study participants were approximately 8 years of age, their mother or guardian was asked to complete a questionnaire item, “On a weekend day, how much time on average does your child spend each day out of doors in summer?” Children were classified as spending a “high” amount of time outdoors if the response was “3 or more hours” and as “low” if the response was “1–2 hours,” “less than 1 hours,” or “not at all.”^25^ In answer to another question on the same questionnaire, “On normal days in school holidays, how much time on average does your child spend each day reading books for pleasure?” children were classified as spending a “high” amount of time reading if the response was “1–2 hours” or “3 or more hours” and as “low” if the response was “less than 1 hours” or “not at all.”^25^ Parental myopia was inferred from a questionnaire item completed by each parent separately during the time the study child's mother was pregnant, which asked, “How would you rate your sight without glasses?” as described previously.^12^ Briefly, parents who responded for both eyes as “I can't see clearly at a distance” or “I can't see much at all” or a combination of these two responses were classed as being myopic. Parents who responded for both eyes as “always very good” or “I can't see clearly close up” or a combination of these two responses were classed as being nonmyopic. Any other combination of responses resulted in the classification being set as “missing.”

#### Generation R

When study participants were approximately 9 years old, their mother or guardian was asked to complete the questionnaire items, “How many days per week does your child play outside?” and “How long does your child approximately play outside per day?”^26^ Mean weekly outdoor play was calculated by multiplying the amount of days × time. Walking or cycling to and from school was processed similarly. Total outdoor exposure was calculated as the sum of playing outside and walking or cycling to and from school. Children were classified as spending a “high” amount of time outdoors if the total weekly outdoor exposure was more than 7 hours and as “low” if the total weekly outdoor exposure was less than 7 hours. In answer to another question on the same questionnaire, “Does your child read in his or her spare time?” children were classified as spending a “high” amount of time reading if the response was “5 to 10 hours per week,” “11 to 15 hours per week,” or “over 15 hours per week” and as “low” if their answer was “never” or “less than 5 hours per week.”^26^ Parental myopia was inferred from the same questionnaire with the items, “Does the mother/father have glasses or contact lenses for either near (minus lenses) or far sightedness (plus lenses)?” Parental myopia was classified as “1” when at least one of the parents was myopic and “0” otherwise.

### Polygenic Risk Scores

Genotype data were available for 7981 ALSPAC participants and 5731 Generation R participants, after excluding individuals who withdrew consent (for details of genotyping and imputation, see Taylor et al.^27^ and Kruithof et al.^24^). Polygenic risk scores were calculated as the weighted number of risk alleles carried for 146 of 149 genetic variants associated with refractive error identified in a genome-wide association study (GWAS) study by the Consortium for Refractive Error and Myopia (CREAM) consortium and 23andMe^8^ (note that 3 of the 149 variants were excluded due to a low minor allele frequency^28^). Weightings were obtained as the regression coefficient for association with refractive error in diopters in the UK Biobank replication sample, as reported by Tedja et al.^8^ Polygenic risk scores were standardized (to have a mean of zero) and then converted to a binary variable, which was coded as “1” if the polygenic risk score was less than zero and coded as “0” otherwise (such that “genetic risk = 1” indicated an increased risk of a more negative refractive error).

### Statistical Analysis

The refractive error of each child was calculated as the average mean spherical equivalent in the two eyes. For ALSPAC, analyses were restricted to unrelated children of European genetic ancestry^12^ who had valid autorefraction information from at least one research clinic visit and whose genotype data passed quality control checks.^27^ For Generation R, analyses were restricted to children with valid cycloplegic refractive error measurement and whose genotype data passed quality control checks.^29^ Because of the smaller sample size, all available Generation R participants were included irrespective of ethnicity or relatedness. Conditional quantile regression models^30^ were fitted with the *quantreg* package in R, with refractive error as the dependent variable and risk factor exposure as an independent variable. Children were stratified into 19 quantiles, ranging from 0.05 (toward myopia) to 0.95 (toward hyperopia). Four established risk factors for myopia were evaluated—high genetic predisposition, parental myopia, high time spent reading, and low time outdoors—with each coded as a binary variable (0 or 1), with 1 indicating a higher risk of myopia. As a control, we evaluated sex as a fifth potential risk factor. In previous work, sex was found to display negligible association with refractive error in the ALSPAC and Generation R samples,^9^^,^^25^ and hence it was of interest to test whether a similar lack of association was observed in quantile regression analyses. The effect associated with each risk factor was evaluated using two approaches: “conventional” univariate OLS linear regression analysis (which assumes the effect of the risk factor is the same in everybody) and univariate quantile regression analysis (which allows the effect of the risk factor to vary depending on where in the refractive error distribution an individual lies). For ALSPAC, separate models were fit for refractive error at age 7, 10, 12, or 15 years. As ALSPAC and Generation R are birth cohort studies, the age range of participants was narrow. Accordingly, the inclusion of a covariate indicating each child's precise age had minimal effect on parameter estimates, and hence an age term was not included. A categorical covariate for self-reported ethnicity was included in the Generation R analyses (self-reported ethnicity was preferred to genetically assessed ethnicity because of a lower level of missing data). Self-reported ethnicity was the only covariate included in the Generation R analyses. No covariates were included in the ALSPAC analyses. The relationship between risk factor effect size and refractive error quantile was modeled using a Loess function with the *ggplot2* package.^31^ Comparisons between the risk factor effect size at a specific quantile versus the risk factor effect size at quantile 0.50 (approximate emmetropia) were assessed by permutation, as described in Appendix 1 in the Supplementary Information. Also, for each risk factor, a test for a linear trend of increasing or decreasing effect size with age in ALSPAC participants was carried out using a random-effects meta-regression model with the *metafor* package.^32^ These trend tests were carried out separately for the effect sizes obtained by OLS regression and by CQR at each quantile. As sensitivity analyses, the primary analyses were repeated after imputing missing data using multiple imputation by chained equations (MICE), as described in Appendix 2 in the Supplementary Information.

## Results

### Cohort Demographics

The age and refractive error of children in the study sample are summarized in Table 1, stratified by research clinic target age. In the ALSPAC sample, after excluding participants with no genetic data, those of non-European genetic ancestry, and those related to other children in the sample, there were 6440 children with refractive error information available from at least one visit (5564, 5291, 4839, and 3687 children had information from the age 7, age 10, age 12, and age 15 research clinics, respectively). Of the full sample, 49.6% were female and 58.9% had one or two parents with myopia, while 41.8%, 20.3%, and 19.5%, were missing information regarding parental myopia, time spent reading, and time spent outdoors, respectively. In the Generation R sample, 2395 participants attended the age 9 research clinic, underwent cycloplegic autorefraction, and had information regarding their ethnicity. Of this sample, 49.9% were female, 67.8% were of European ethnicity, and 53% had one or two parents with myopia, while 40.0%, 35.8%, 34.4%, and 18.0% were missing information regarding genotypes, parental myopia, time spent reading, and time spent outdoors, respectively (Table 1).

**Table 1.** Demographic Characteristics of Children With Information Available at Each Research Clinic Visit

Research Clinic	*N*	Sex* (Boys/Girls)	Age,^†^ y	Ethnicity(European/non-European)	Refractive Error^†^(D)	Genetic Risk* Low/High/Missing, No. (%)	Parental myopia*No/Yes/Missing, No. (%)	Time Reading*Low/High/Missing, No. (%)	Time Outdoors* Low/High/Missing,No. (%)
**ALSPAC**									
Age 7	5564	2827/2737	7.53(6.92 to 8.13)	5564/0	0.20(–1.53 to 1.92)	2772/2792/0(49.8/50.2/0.0)	1341/1979/2244(24.1/35.6/40.3)	2923/1700/941(52.5/30.6/16.9)	2210/2450/904(39.7/44.0/16.2)
Age 10	5291	2622/2669	10.64 (10.15 to 11.13)	5291/0	0.06(–2.06 to 2.19)	2645/2646/0(50.0/50.0/0.0)	1269/1898/2124(24.0/35.9/40.1)	2801/1679/811(52.9/31.7/15.3)	2142/2376/773(40.5/44.9/14.6)
Age 12	4839	2360/2479	12.81 (12.36 to 13.25)	4839/0	–0.16(–2.45 to 2.12)	2423/2416/0(50.1/49.9/0.0)	1154/1747/1938(23.8/36.1/40.0)	2554/1544/741(52.8/31.9/15.3)	1937/2195/707(40.0/45.4/14.6)
Age 15	3687	1745/1942	15.43(14.89 to 15.97)	3687/0	–0.38(–2.89 to 2.12)	1850/1837/0(50.2/49.8/0.0)	886/1387/1414(24.0/37.6/38.4)	1958/1251/478(53.1/33.9/13.0)	1449/1779/459(39.3/48.3/12.4)
**Generation R**									
Age 9	2395	1200/1195	9.83(9.82 to 9.85)	1625/770	0.75(0.70 to 0.80)	718/720/957(30.0/30.0/40.0)	718/820/857(30.0/34.2/35.8)	973/599/823(40.6/25.0/34.4)	995/968/432(41.5/40.4/18.0)

* Sample size in each category.

† Mean (95% CI).

### Association Between Risk Factor Exposure and Refractive Error

**Figure 1.**
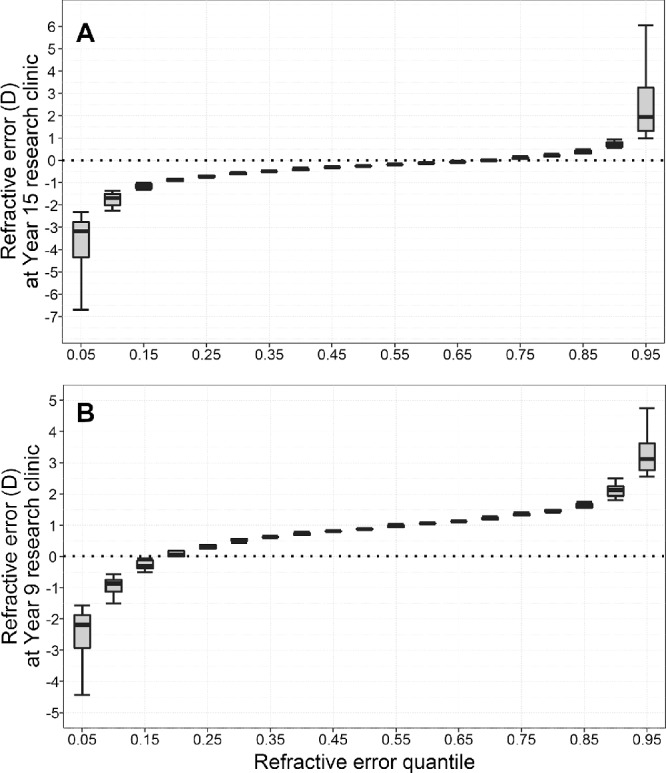
Distribution of refractive error by quantiles. (**A**) refractive error at the age 15 research clinic in ALSPAC participants. (**B**) Refractive error at the age 9 research clinic in Generation R participants. Participants in each study sample were ranked by refractive error (most myopic to most hyperopic) and then divided into 19 equally sized bins (quantiles).

**Figure 2.**
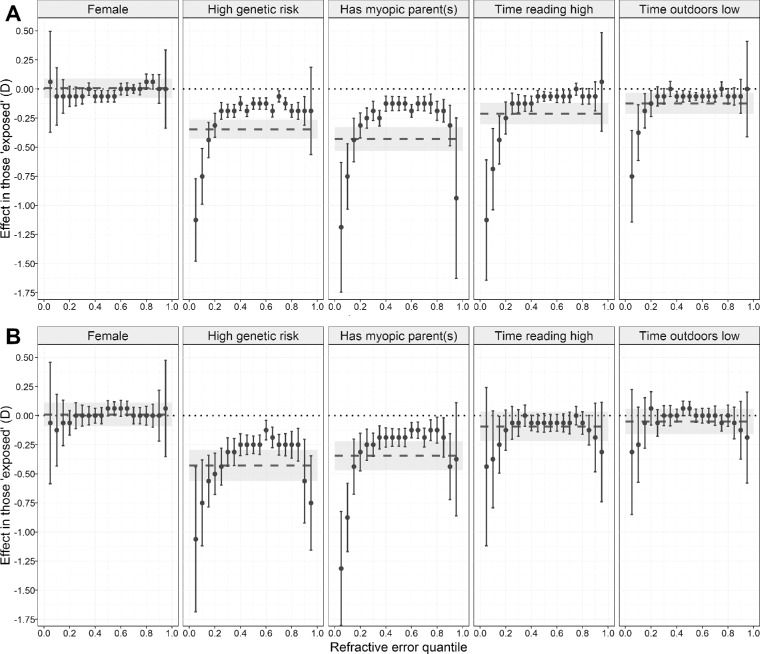
Comparison of effect sizes associated with risk factor exposure estimated with OLS linear regression or with quantile regression. (**A**) Refractive error at the age 15 research clinic in ALSPAC participants. (**B**) Refractive error at the age 9 research clinic in Generation R participants. The *dashed line* indicates the effect size associated with exposure to the risk factor, calculated with OLS linear regression (95% confidence interval shown as *gray shaded region*). *Filled circles* correspond to the effect size associated with each exposure, calculated with quantile regression (error bars indicate 95% confidence interval). Note that effect sizes can vary across quantiles of the refractive error distribution for quantile regression.

Figure 1 illustrates how refractive error was distributed across quantiles of the trait in ALSPAC participants attending the age 15 research clinic and Generation R participants attending the age 9 research clinic. In the ALSPAC sample, conventional OLS regression analysis provided evidence that four of the five risk factors were associated with a more negative refractive error: a high genetic risk, having a parent with myopia, a high amount of time spent reading, and a low amount of time spent outdoors (Table 2). Sex showed little evidence of an association with refractive error in this sample, although there was weak evidence of an association at age 12 (*β* = –0.07 D, 95% CI –0.14 to –0.00 D, *P* = 0.040 for females). The effect associated with the other four risk factors steadily increased in magnitude as children got older (e.g., the effect size was –0.06, –0.10, –0.15, and –0.21 D at the age 7, 10, 12, and 15 research clinics, respectively, in participants who spent a high versus low time reading). In the Generation R sample, conventional OLS regression analysis provided evidence that two of the five risk factors were associated with a more negative refractive error: a high genetic risk (*β* = –0.43 D, 95% CI –0.56 to –0.30, *P* < 0.001) and having a parent with myopia (*β* = –0.35 D, 95% CI –0.47 to –0.22, *P* < 0.001) (Table 2). The effect associated with each risk factor in ALSPAC individuals attending the age 15 research clinic and Generation R individuals attending the age 9 research clinic is shown in Figure 2 (as a dashed line, with 95% confidence interval depicted with gray shading).

**Table 2.** Effect Sizes Quantifying Associations Between Risk Factors and Refractive Error, Evaluated Using OLS Linear Regression or Quantile Regression

Risk Factor	Cohort	Research Clinic	*N*	OLS Regression		Quantile Regression: Quantile 0.05		Quantile Regression: Quantile 0.50	
				*β* (95% CI)	*P* Value	*β* (95% CI)	*P* Value	*β* (95% CI)	*P* Value
Female sex	ALSPAC	Age 7	5564	–0.016 (–0.062 to 0.030)	4.92E–01	0.000 (–0.090 to 0.090)	1.00E+00	0.000 (–0.030 to 0.030)	1.00E+00
		Age 10	5291	0.009 (–0.050 to 0.067)	7.66E–01	0.062 (–0.118 to 0.243)	4.98E–01	0.000 (–0.039 to 0.039)	1.00E+00
		Age 12	4839	–0.069 (–0.135 to –0.003)	3.98E–02	–0.188 (–0.448 to 0.073)	1.59E–01	0.000 (–0.031 to 0.031)	1.00E+00
		Age 15	3687	0.008 (–0.075 to 0.090)	8.54E–01	0.062 (–0.371 to 0.496)	7.78E–01	–0.062 (–0.111 to –0.014)	1.11E–02
	Generation R	Age 9	2395	0.010 (–0.091 to 0.111)	8.47E–01	–0.063 (–0.585 to 0.460)	8.15E–01	0.063 (–0.003 to 0.128)	6.21E–02
High genetic risk	ALSPAC	Age 7	5564	–0.163 (–0.209 to –0.117)	3.91E–12	–0.188 (–0.282 to –0.093)	1.00E–04	–0.125 (–0.155 to –0.095)	4.44E–16
		Age 10	5291	–0.235 (–0.293 to –0.177)	2.28E–15	–0.438 (–0.634 to –0.241)	1.30E–05	–0.125 (–0.155 to –0.095)	6.66E–16
		Age 12	4839	–0.300 (–0.365 to –0.235)	2.73E–19	–0.812 (–1.084 to –0.541)	4.54E–09	–0.125 (–0.156 to –0.094)	1.78E–15
		Age 15	3687	–0.345 (–0.427 to –0.264)	1.53E–16	–1.125 (–1.479 to –0.771)	5.01E–10	–0.125 (–0.173 to –0.077)	3.73E–07
	Generation R	Age 9	1437	–0.428 (–0.561 to –0.295)	4.01E–10	–1.063 (–1.685 to –0.440)	8.43E–04	–0.250 (–0.325 to –0.175)	9.68E–11
Has myopic parent(s)	ALSPAC	Age 7	3320	–0.257 (–0.313 to –0.202)	2.07E–19	–0.250 (–0.364 to –0.136)	1.81E–05	–0.125 (–0.158 to –0.092)	2.22E–13
		Age 10	3167	–0.350 (–0.423 to –0.278)	4.20E–21	–0.688 (–0.886 to –0.489)	1.45E–11	–0.125 (–0.169 to –0.081)	3.70E–08
		Age 12	2901	–0.388 (–0.470 to –0.306)	4.05E–20	–1.000 (–1.385 to –0.615)	3.80E–07	–0.125 (–0.167 to –0.083)	4.84E–09
		Age 15	2273	–0.430 (–0.533 to –0.327)	4.38E–16	–1.188 (–1.745 to –0.630)	3.10E–05	–0.125 (–0.186 to –0.064)	6.18E–05
	Generation R	Age 9	1537	–0.345 (–0.468 to –0.221)	5.69E–08	–1.313 (–1.801 to –0.824)	1.60E–07	–0.188 (–0.262 to –0.113)	1.02E–06
Time spent reading high	ALSPAC	Age 7	4623	–0.062 (–0.114 to –0.011)	1.77E–02	–0.188 (–0.327 to –0.048)	8.57E–03	0.000 (–0.032 to 0.032)	1.00E+00
		Age 10	4480	–0.104 (–0.170 to –0.038)	2.09E–03	–0.438 (–0.672 to –0.203)	2.52E–04	0.000 (–0.039 to 0.039)	1.00E+00
		Age 12	4098	–0.153 (–0.227 to –0.079)	4.86E–05	–0.750 (–1.111 to –0.389)	4.75E–05	–0.062 (–0.106 to –0.019)	4.77E–03
		Age 15	3209	–0.211 (–0.303 to –0.119)	7.04E–06	–1.125 (–1.642 to –0.608)	2.03E–05	–0.062 (–0.104 to –0.021)	2.86E–03
	Generation R	Age 9	1572	–0.092 (–0.220 to 0.035)	1.56E–01	–0.438 (–1.117 to 0.242)	2.07E–01	–0.063 (–0.142 to 0.017)	1.26E–01
Time spent outdoors low	ALSPAC	Age 7	4660	–0.051 (–0.100 to –0.001)	4.60E–02	–0.062 (–0.140 to 0.015)	1.15E–01	0.000 (–0.031 to 0.031)	1.00E+00
		Age 10	4518	–0.097 (–0.161 to –0.033)	3.01E–03	–0.312 (–0.514 to –0.111)	2.34E–03	0.000 (–0.040 to 0.040)	1.00E+00
		Age 12	4132	–0.122 (–0.194 to –0.051)	8.06E–04	–0.562 (–0.849 to –0.276)	1.19E–04	0.000 (–0.040 to 0.040)	1.00E+00
		Age 15	3228	–0.123 (–0.213 to –0.034)	7.15E–03	–0.750 (–1.143 to –0.357)	1.85E–04	–0.062 (–0.105 to –0.020)	4.28E–03
	Generation R	Age 9	1963	–0.051 (–0.160 to 0.058)	3.63E–01	–0.313 (–0.850 to 0.225)	2.54E–01	0.063 (0.003 to 0.122)	4.06E–02

All risk factors were coded as binary variables (0 = lower risk and 1 = higher risk category for myopia). Results for quantile regression are only presented for quantiles 0.05 and 0.50. The sample size at each age varies due to missing information for some risk factors.

Quantile regression analysis also suggested that the same four risk factors in the ALSPAC sample and the same two risk factors in the Generation R sample identified by OLS regression were associated with refractive error (Table 2; Supplementary Tables S1 and S2). In addition, this method yielded compelling evidence that the effect associated with exposure to each of these risk factors varied markedly between individuals (Fig. 2; Supplementary Tables S1 and S2). The pattern of results was similar for the genetic risk score and for having a myopic parent. Namely, the effect size was of the order of –0.13 D for participants who were in the middle of the trait distribution (i.e., emmetropes and low ametropes) while the estimated effect size was increasingly larger for children in the more extreme quantiles. For example, the increased risk associated with having at least one myopic parent was seven to nine times larger for children in quantile 0.05 versus quantile 0.50 in ALSPAC children aged 15 years (–1.19 D, 95% CI –1.75 to –0.63 D vs. –0.13 D, 95% CI –0.19 to –0.06 D, *P* = 0.001) as well as in Generation R children aged 9 years (–1.31 D, 95% CI –1.80 to –0.82 D vs. –0.19 D, 95% CI –0.26 to –0.11 D, *P* < 0.001) (Table 2; Supplementary Table S1). For time spent outdoors and time spent reading in the ALSPAC sample, the effect associated with the risk factor was very close to zero for children in quantiles 0.50 (approximate emmetropia) to 0.95 (hyperopia), while the effect size estimates became increasingly more negative for progressively lower quantiles (myopia). For the lowest quantile (0.05), the estimated effect size associated with a “high” time reading was –1.13 D and the effect size associated with a “low” time outdoors was –0.75 D (*P* < 0.001 and *P* = 0.001, respectively, for the comparison between quantile 0.05 vs. 0.50; Supplementary Table S1). There was minimal evidence of an association between refractive error and either time spent outdoors or time spent reading in the Generation R sample at any quantile, mirroring the OLS analysis results. Sensitivity analyses carried out after imputing missing data yielded similar effect size estimates to the original analyses but with more precise confidence intervals (Supplementary Table S3 and Figure S1). This led to stronger support for an association of time spent reading and refractive error in the Generation R sample after imputation of missing data (*P* = 0.031 in OLS analysis and *P* = 0.001 for CQR at quantile 0.05; Supplementary Table S3).

**Figure 3.**
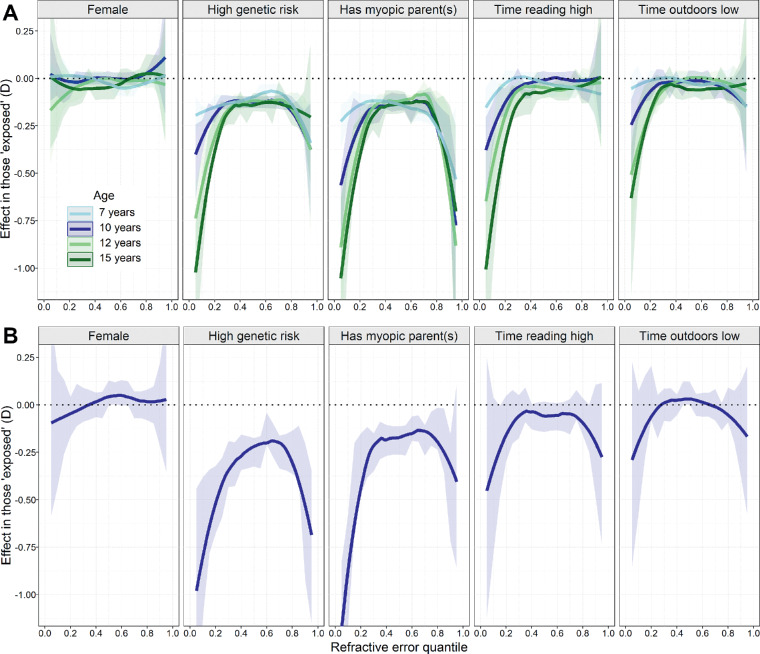
Pattern of effect sizes associated with risk factor exposure estimated with quantile regression. (**A**) Refractive error at the age 7 to age 15 research clinics in ALSPAC participants. (**B**) Refractive error at the age 9 research clinic in Generation R participants. The *fitted lines* indicate the effect size associated with exposure to the risk factor (*shaded regions* indicate 95% confidence interval of Loess fit).

Finally, quantile regression analysis was used to track the change in effect size associated with each risk factor across childhood in the ALSPAC sample (Fig. 3A). There was evidence that children in the high genetic risk group and children with at least one myopic parent already had a more negative refractive error at age 7 years. This was true even for individuals in the middle of the trait distribution (e.g., at quantile 0.50, genetic risk *β* = –0.13 D, *P* < 0.001 and parental myopia *β* = –0.13 D, *P* < 0.001). This was not the case for time spent reading and time outdoors at age 7 years (at quantile 0.50, time reading *β* = 0.00 D, *P* = 1.00 and parental myopia *β* = 0.00 D, *P* = 1.00). For all risk factors except sex, effect sizes steadily increased with age (Fig. 3A; Table 2). Tests for a linear trend of increasing effect size with age revealed statistical evidence supporting such increases for all risk factors except sex across quantiles 0.05 to 0.20 (Supplementary Table S4). However, there was no evidence to suggest that effect sizes increased linearly with age for children in the middle and higher quantiles (quantiles 0.50 to 0.95). The pattern of results in Generation R at age 9 (Fig. 3B; Table 2) was broadly similar to that of ALSPAC children at age 10. Sensitivity analyses after imputing missing data yielded similar results (Supplementary Figure S2).

## Discussion

In the ALSPAC and Generation R birth cohorts, we observed evidence that both genetic and environmental risk factors were associated with large, interindividual variations in effect on refractive error. In other words, the effect of being exposed to one of the risk factors was not the same for all children. This interindividual variation was not apparent when conventional OLS linear regression was used. In the main analyses, parental myopia and the genetic risk score were associated with refractive error in both ALSPAC and Generation R. However, reading time and time outdoors were associated with refractive error in ALSPAC but not in the Generation R cohort. The absence of an association in Generation R for the two environmental risk factors may be due to limited power, since the *β*-coefficients for near work and outdoor exposure in Generation R were very similar to the ALSPAC age 7 cohort, but the sample size was smaller, leading to lower precision. In support of this theory, analysis of the Generation R sample using multiple imputation of missing data *did* provide evidence of an association with near work (OLS analysis, *β* = –0.29 D, 95% CI –0.53 to –0.04, *P* = 0.031; CQR analysis for quantile 0.05, *β* = –0.86 D, 95% CI –1.41 to –0.32, *P* = 0.001). Moreover, these environmental risk factors have previously been associated with myopia and axial elongation in a larger sample from Generation R when the exposures were modeled as continuous variables.^9^^,^^33^ Our results highlighted differences in effect size profiles for genetic and environmental factors. Most evidently, genetic risk and parental myopia were associated with refractive error in children from both the myopic and the hyperopic arms of the refractive error distribution, whereas environmental factors were only associated with refractive error in children in the myopic arm of the distribution, tentatively suggesting that myopia may be both genetically and environmentally driven, while hyperopia may be only genetically driven. In contrast, sex showed little variation in effects across different quantiles, and the CQR effect size estimate was comparable to that obtained by OLS linear regression. It was not possible to determine whether effect sizes were larger for genetic than for environmental risk factors, because the risk factors would have been measured with varying degrees of imprecision and error (e.g., a parental questionnaire is known to be a crude method of quantifying time spent outdoors^34^).

Further analysis stratifying children by age suggested that effects on refractive error associated with the risk exposures were not fixed. Instead, there was evidence for a monotonic increase in genetic and environmental effect size estimates with additional years of age, restricted to quantiles 0.05 to 0.30. The difference between the youngest (7 years) and the oldest (15 years) ALSPAC participants was most pronounced for children in quantile 0.05 (i.e., those with the most myopic refractive error) and reached as much as 0.93 D. Our OLS analyses also showed an increased effect with age for all risk factors except sex (Supplementary Table S4), although the evidence was weaker for time spent outdoors (*P* = 0.07) than for the other risk factors (all *P* ≤ 0.002). Age-dependent effects have been established in previous studies regarding genetics; for example, it has been reported that specific genetic variants may have “early” or “late” effects.^35^^,^^36^ Regarding environmental effects, a meta-analysis of outdoor exposure stratified by age showed conflicting results, while the effect of near work stratified by age has not been studied in detail.^10^^,^^11^

To our knowledge, this is the first study investigating environmental risk factors for myopia using quantile regression. As regards genetic risk, a recent quantile regression study proposed visually guided emmetropization to be the mechanism by which effect size heterogeneity arises.^28^ Emmetropization is a process that is influenced by both genetic and environmental (visual) factors.^37^ Mutti et al.^38^ have proposed that emmetropizatory lens thinning has a limit to the amount of axial elongation that it can compensate for. Given the age range of our study population, we extend this hypothesis and suggest that emmetropization might have a protective effect not only against myopia- or hyperopia-predisposing genetic risk factors but also myopia-inducing environmental risk factors such as time spent reading or time spent outdoors. Together with our finding that the effects of genetic and environmental risk factors increase with age, we hypothesize that for those individuals whose emmetropization compensation limit is surpassed, genetic and environmental risk factors could lead to greater effects.

Gene-environment interactions have been identified in adult and child population using genetic risk scores and education or an environmental sum score.^9^^,^^39^ Analyses of the adult and child populations from CREAM resulted in only a few gene-environment interactions using individual genetic variants and education or near work.^36^^,^^40^^,^^41^ In the absence of interactions (such as gene-environment interactions), all individuals would be expected to respond to risk factors in the same way. However, our analysis identified a high degree of variability from person to person, hinting toward potential involvement of gene-environment or other kinds of interaction (i.e., gene-gene or environment-environment interactions). For example, an individual's genetic risk remains essentially fixed during the lifetime, and yet we see effect size heterogeneity not only for one age category (e.g., age 15) but across different age groups. Therefore, we suggest that genetic effects on refractive error may depend on the amount of time an individual has been exposed to an environmental risk factor. Therefore, lifestyle changes may be particularly beneficial for children destined to reach the extreme myopic arm of the refractive error distribution by adulthood (although identifying such children prior to myopia development is challenging^42^^,^^43^). Myopia control interventions, such as atropine eye drops or orthokeratology, may be particularly beneficial in these children.^44^^,^^45^ With reference to the various treatment interventions for myopia, it has been reported that certain children respond particularly well to a specific intervention while others respond poorly.^44^ This is reminiscent of the interindividual variation in risk factor effect sizes revealed here by quantile regression analysis. Thus, we propose that quantile regression analysis of clinical trial data may be an informative future direction for research aimed at better understanding the causes of interindividual treatment responses.

A strength of this study is the triangulation of research methods. Both OLS and CQR analyses were performed in two cohorts to minimize bias and to strengthen our conclusions. Furthermore, in the ALSPAC cohort, noncycloplegic refractive error measurements were performed up to five times from age 7 to age 15, which allowed us to analyze patterns over childhood. Because of the large sample size of the ALSPAC cohort, we had the opportunity to exclude children of non-European ethnicity and familially related children to ensure that these factors did not influence our findings. The use of cycloplegia in the Generation R cohort made this cohort ideal as a replication sample to investigate the impact of the absence of cycloplegia in ALSPAC. Unfortunately, the smaller sample size of Generation R necessitated that children of non-European ethnicity and related children were *not* excluded. We were limited by the use of questionnaire data for near work, outdoor exposure, and parental myopia, which may have influenced our results because of coarse-grained response options and errors in gauging the duration of children's past behavior by parents. Furthermore, the criteria used to define time spent outdoors and time spent reading as being either “high” or “low” differed between ALSPAC and Generation R. It was not possible to standardize classification criteria across the two studies since ALSPAC and Generation R utilized different questions and response options to gauge time outdoors and time reading. The classification criteria we adopted were, in general, those employed in previous investigations of risk factors for myopia.^25^^,^^26^^,^^36^ The exception to this was time outdoors in the Generation R study, which was previously modeled as a continuous variable^26^^,^^33^ but here was dichotomized to split the sample into two groups of approximately equal size. Analyses of the two cohorts also differed regarding the ethnicity of the participants: all ALSPAC participants were of European ancestry, while approximately 32% of Generation R participants were of non-European ancestry. The inclusion of children with diverse ethnic backgrounds may have increased effect size estimates in the Generation R cohort relative to ALSPAC if, as has previously been suggested, myopia risk factor effect sizes are larger in children of non-European ethnicity.^40^

We were also limited by the high level of missing data for both cohorts, especially for the risk factors derived from questionnaire responses (time spent outdoors, time spent reading, and parental myopia). Sensitivity analyses carried out after imputing missing data provided comparable results to the original analyses, suggesting that the high level of missing data would have had little impact so long as these data were missing at random. Should the data not have been missing at random, this could have biased both the OLS and CQR effect size estimates. A third limitation was that refractive error in the ALSPAC cohort was assessed without cycloplegia. A comparison of noncycloplegic autorefraction and cycloplegic retinoscopy in ALSPAC children who had pinhole visual acuity >0.2 logMAR at age 7 (*n* = 414) revealed an average discrepancy of –0.13 D (standard deviation 0.53 D).^46^ At age 15, a comparison of noncycloplegic autorefraction and optometrist spectacle prescriptions in ALSPAC participants (*n* = 346 individuals with data available from the age 15 clinic visit and an optometrist spectacle prescription within ±6 months) yielded an average error of –0.22 D (standard deviation 0.84 D).^47^ At both ages, the negative bias in estimates of refractive error due to lack of cycloplegia was greater in those with hyperopia than those with myopia, as reported previously.^48^ Therefore, this source of measurement error could have affected the estimation of risk factor effect sizes at certain quantiles more than at other quantiles. Specifically, if the higher quantiles (comprising children in the hyperopic arm of the refractive error distribution) were relatively more affected by measurement error, then this may have led to the attenuation of risk factor effect size estimates for these higher quantiles. This phenomenon would in turn have attenuated the difference in effect size between high versus middle quantiles, for example, quantiles 0.95 vs. 0.50 (although we caution that the effects of measurement error can be difficult to predict^49^). Notably, the inverted U-shape of the risk factor effect size versus quantile relationship was apparent for both the ALSPAC and Generation R cohorts, which provided reassurance that measurement error resulting from lack of cycloplegia was not a major contributor to the this inverted U-shaped relationship. Cycloplegic refractive error measurements were introduced 1.5 years after the start of the Generation R age 9 research clinic. We restricted our analysis to the 2395 children who received cycloplegia. We expect this selection process of excluding children who did not undergo cycloplegic autorefraction not to have introduced bias but rather to have reduced the statistical power of the analyses. Finally, we chose to examine binary risk factors in order to simplify interpretation, but this may also have led to reduced statistical power in our models.

In conclusion, quantile regression analysis of two large, population-based birth cohorts provided evidence that both genetic and environmental risk factors for myopia have widely differing impacts in different individuals (e.g., sevenfold or more). Our findings are consistent with the idea that each person's final position in the refractive error distribution is the result of not only his or her level of genetic risk and exposure to environmental risk factors but also his or her emmetropization system's ability to buffer against these risk factors.

## Supplementary Material

Supplement 1

## References

[1] Williams KM, Bertelsen G, Cumberland P, et al. Increasing prevalence of myopia in Europe and the impact of education. *Ophthalmology*. 2015; 122: 1489–1497. 25983215 10.1016/j.ophtha.2015.03.018 PMC4504030

[2] Wei S, Sun Y, Li S, et al. Refractive errors in university students in central China: the Anyang University Students Eye Study. *Invest Ophthalmol Vis Sci*. 2018; 59: 4691–4700. 30267091 10.1167/iovs.18-24363

[3] Verkicharla PK, Ohno-Matsui K, Saw SM Current and predicted demographics of high myopia and an update of its associated pathological changes. *Ophthalmic Physiol Opt*. 2015; 35: 465–475. 26303444 10.1111/opo.12238

[4] Wong TY, Ferreira A, Hughes R, Carter G, Mitchell P Epidemiology and disease burden of pathologic myopia and myopic choroidal neovascularization: an evidence-based systematic review. *Am J Ophthalmol*. 2014; 157: 9–25. 24099276 10.1016/j.ajo.2013.08.010

[5] Wong YL, Sabanayagam C, Ding Y, et al. Prevalence, risk factors, and impact of myopic macular degeneration on visual impairment and functioning among adults in Singapore. *Invest Ophthalmol Vis Sci*. 2018; 59: 4603–4613. 30242361 10.1167/iovs.18-24032

[6] Verhoeven VJ, Wong KT, Buitendijk GH, Hofman A, Vingerling JR, Klaver CC Visual consequences of refractive errors in the general population. *Ophthalmology*. 2015; 122: 101–109. 25208857 10.1016/j.ophtha.2014.07.030

[7] Schaeffel F, Feldkaemper M Animal models in myopia research. *Clin Exp Optom*. 2015; 98: 507–517. 26769177 10.1111/cxo.12312

[8] Tedja MS, Wojciechowski R, Hysi PG, et al. Genome-wide association meta-analysis highlights light-induced signaling as a driver for refractive error. *Nat Genet*. 2018; 50: 834–848. 29808027 10.1038/s41588-018-0127-7 PMC5980758

[9] Enthoven CA, Tideman JWL, Polling JR, et al. Interaction between lifestyle and genetic susceptibility in myopia: the Generation R study. *Eur J Epidemiol*. 2019; 34: 777–784. 30945054 10.1007/s10654-019-00512-7 PMC6602996

[10] Huang HM, Chang DS, Wu PC The association between near work activities and myopia in children: a systematic review and meta-analysis. *PLoS One*. 2015; 10: e0140419. 26485393 10.1371/journal.pone.0140419 PMC4618477

[11] Xiong S, Sankaridurg P, Naduvilath T, et al. Time spent in outdoor activities in relation to myopia prevention and control: a meta-analysis and systematic review. *Acta Ophthalmol*. 2017; 95: 551–566. 28251836 10.1111/aos.13403 PMC5599950

[12] Ghorbani Mojarrad N, Williams C, Guggenheim JA A genetic risk score and number of myopic parents independently predict myopia. *Ophthalmic Physiol Opt*. 2018; 38: 492–502. 30182516 10.1111/opo.12579

[13] Lin LL, Shih YF, Tsai CB, et al. Epidemiologic study of ocular refraction among schoolchildren in Taiwan in 1995. *Optom Vis Sci*. 1999; 76: 275–281. 10375241 10.1097/00006324-199905000-00013

[14] Vitale S, Ellwein L, Cotch MF, Ferris FL III, Sperduto R Prevalence of refractive error in the United States, 1999–2004. *Arch Ophthalmol*. 2008; 126: 1111–1119. 18695106 10.1001/archopht.126.8.1111 PMC2772054

[15] Yip VC, Pan CW, Lin XY, et al. The relationship between growth spurts and myopia in Singapore children. *Invest Ophthalmol Vis Sci*. 2012; 53: 7961–7966. 23150611 10.1167/iovs.12-10402

[16] Beyerlein A Quantile regression—opportunities and challenges from a user's perspective. *Am J Epidemiol*. 2014; 180: 330–331. 24989240 10.1093/aje/kwu178

[17] Azagba S, Sharaf MF Fruit and vegetable consumption and body mass index: a quantile regression approach. *J Prim Care Community Health*. 2012; 3: 210–220. 23803782 10.1177/2150131911434206

[18] Beyerlein A, Fahrmeir L, Mansmann U, Toschke AM Alternative regression models to assess increase in childhood BMI. *BMC Med Res Methodol*. 2008; 8: 59. 18778466 10.1186/1471-2288-8-59 PMC2543035

[19] Williams PT Quantile-specific penetrance of genes affecting lipoproteins, adiposity and height. *PLoS One*. 2012; 7: e28764. 22235250 10.1371/journal.pone.0028764 PMC3250394

[20] Burgette LF, Reiter JP, Miranda ML Exploratory quantile regression with many covariates: an application to adverse birth outcomes. *Epidemiology*. 2011; 22: 859–866. 21968775 10.1097/EDE.0b013e31822908b3

[21] Boyd A, Golding J, Macleod J, et al. Cohort profile: the 'children of the 90s'—the index offspring of the Avon Longitudinal Study of Parents and Children. *Int J Epidemiol*. 2013; 42: 111–127. 22507743 10.1093/ije/dys064 PMC3600618

[22] Fraser A, Macdonald-Wallis C, Tilling K, et al. Cohort profile: the Avon Longitudinal Study of Parents and Children: ALSPAC mothers cohort. *Int J Epidemiol*. 2012; 42: 97–110. 22507742 10.1093/ije/dys066 PMC3600619

[23] Kooijman MN, Kruithof CJ, van Duijn CM, et al. The Generation R Study: design and cohort update 2017. *Eur J Epidemiol*. 2016; 31: 1243–1264. 28070760 10.1007/s10654-016-0224-9 PMC5233749

[24] Kruithof CJ, Kooijman MN, van Duijn CM, et al. The Generation R study: Biobank update 2015. *Eur J Epidemiol*. 2014; 29: 911–927. 25527369 10.1007/s10654-014-9980-6

[25] Guggenheim JA, Northstone K, McMahon G, et al. Time outdoors and physical activity as predictors of incident myopia in childhood: a prospective cohort study. *Invest Ophthalmol Vis Sci*. 2012; 53: 2856–2865. 22491403 10.1167/iovs.11-9091 PMC3367471

[26] Tideman JWL, Polling JR, Hofman A, Jaddoe VW, Mackenbach JP, Klaver CC Environmental factors explain socioeconomic prevalence differences in myopia in 6-year-old children. *Br J Ophthalmol*. 2018; 102: 243–247. 28607175 10.1136/bjophthalmol-2017-310292

[27] Taylor M, Simpkin AJ, Haycock PC, Dudbridge F, Zuccolo L Exploration of a polygenic risk score for alcohol consumption: a longitudinal analysis from the ALSPAC cohort. *PLoS One*. 2016; 11: e0167360. 27902751 10.1371/journal.pone.0167360 PMC5130278

[28] Pozarickij A, Williams C, Hysi PG, Guggenheim JA; U.K. Biobank Eye and Vision Consortium. Quantile regression analysis reveals widespread evidence for gene-environment or gene-gene interactions in myopia development. *Commun Biol*. 2019; 2: 167. 31069276 10.1038/s42003-019-0387-5 PMC6502837

[29] Medina-Gomez C, Felix JF, Estrada K, et al. Challenges in conducting genome-wide association studies in highly admixed multi-ethnic populations: the Generation R Study. *Eur J Epidemiol*. 2015; 30: 317–330. 25762173 10.1007/s10654-015-9998-4 PMC4385148

[30] Koenker R, Hallock KF Quantile regression. *J Econ Perspect*. 2001; 15: 143–156.

[31] Wickham H *ggplot2: Elegant Graphics for Data Analysis*. New York, NY: Springer-Verlag; 2016.

[32] Viechtbauer W Conducting meta-analyses in R with the metafor package. *J Stat Softw*. 2010; 36: 1–48.

[33] Tideman JWL, Polling JR, Jaddoe VWV, Vingerling JR, Klaver CCW Environmental risk factors can reduce axial length elongation and myopia incidence in 6- to 9-year-old children. *Ophthalmology**.* 2019; 126: 127–136. 30146089 10.1016/j.ophtha.2018.06.029

[34] Read SA, Collins MJ, Vincent SJ Light exposure and eye growth in childhood. *Invest Ophthalmol Vis Sci*. 2015; 56: 6779–6787. 26567790 10.1167/iovs.14-15978

[35] Tideman JW, Fan Q, Polling JR, et al. When do myopia genes have their effect? Comparison of genetic risks between children and adults. *Genet Epidemiol*. 2016; 40: 756–766. 27611182 10.1002/gepi.21999

[36] Fan Q, Guo X, Tideman JW, et al. Childhood gene-environment interactions and age-dependent effects of genetic variants associated with refractive error and myopia: the CREAM Consortium. *Sci Rep*. 2016; 6: 25853. 27174397 10.1038/srep25853 PMC4865831

[37] Chen YP, Hocking PM, Wang L, et al. Selective breeding for susceptibility to myopia reveals a gene-environment interaction. *Invest Ophthalmol Vis Sci*. 2011; 52: 4003–4011. 21436268 10.1167/iovs.10-7044

[38] Mutti DO, Mitchell GL, Sinnott LT, et al. Corneal and crystalline lens dimensions before and after myopia onset. *Optom Vis Sci*. 2012; 89: 251–262. 22227914 10.1097/OPX.0b013e3182418213 PMC3288626

[39] Verhoeven VJ, Buitendijk GH, Rivadeneira F, et al. Education influences the role of genetics in myopia. *Eur J Epidemiol*. 2013; 28: 973–980. 24142238 10.1007/s10654-013-9856-1 PMC3898347

[40] Fan Q, Verhoeven VJM, Wojciechowski R, et al. Meta-analysis of gene-environment-wide association scans accounting for education level identifies additional loci for refractive error. *Nat Commun*. 2016; 7: 11008. 27020472 10.1038/ncomms11008 PMC4820539

[41] Fan Q, Wojciechowski R, Ikram MK, et al. Education influences the association between genetic variants and refractive error: a meta-analysis of five Singapore studies. *Hum Mol Genet*. 2014; 23: 546–554. 24014484 10.1093/hmg/ddt431 PMC3869359

[42] Zadnik K, Sinnott LT, Cotter SA, et al. Prediction of juvenile-onset myopia. *JAMA Ophthalmol*. 2015; 133: 683–689. 25837970 10.1001/jamaophthalmol.2015.0471 PMC4607030

[43] Ghorbani Mojarrad N, Plotnikov D, Williams C, Guggenheim JA; U.K. Biobank Eye & Vision Consortium. Association between polygenic risk score and risk of myopia [published online ahead of print October 31, 2019]. *JAMA Ophthalmol*. 2019; 10.1001/jamaophthalmol.2019.4421. PMC6824229 31670792

[44] Wildsoet CF, Chia A, Cho P, et al. IMI—interventions for controlling myopia onset and progression report. *Invest Ophthalmol Vis Sci*. 2019; 60: M106–M131. 30817829 10.1167/iovs.18-25958

[45] Prousali E, Haidich AB, Fontalis A, Ziakas N, Brazitikos P, Mataftsi A Efficacy and safety of interventions to control myopia progression in children: an overview of systematic reviews and meta-analyses. *BMC Ophthalmol*. 2019; 19: 106. 31072389 10.1186/s12886-019-1112-3 PMC6506938

[46] Williams C, Miller L, Northstone K, Sparrow JM The use of non-cycloplegic autorefraction data in general studies of children's development. *Br J Ophthalmol*. 2008; 92: 723–724. 18441189 10.1136/bjo.2007.136051

[47] Northstone K, Guggenheim JA, Howe LD, et al. Body stature growth trajectory during childhood and the development of myopia. *Ophthalmology*. 2013; 120: 1064–1073. 23415774 10.1016/j.ophtha.2012.11.004 PMC4441725

[48] Zhao J, Mao J, Luo R, Li F, Pokharel GP, Ellwein LB Accuracy of noncycloplegic autorefraction in school-age children in China. *Optom Vis Sci*. 2004; 81: 49–55. 14747761 10.1097/00006324-200401000-00010

[49] Kipnis V, Freedman LS. Impact of exposure measurement error in nutritional epidemiology. *J Natl Cancer Inst*. 2008; 100: 1658–1659. 19033567 10.1093/jnci/djn408

