# Supplementary material for: Evidence That Emmetropization Buffers Against Both Genetic and Environmental Risk Factors for Myopia
Source: Invest Ophthalmol Vis Sci. 2020 Feb 25;61(2):41. doi: 10.1167/iovs.61.2.41 (PMC7329625; doi:10.1167/iovs.61.2.41)
Supplement: Supplement 1 [file iovs-61-2-41_s001.pdf]

# Evidence that emmetropization buffers against both genetic and environmental risk factors for myopia

## Supplementary information


| Item | Title | Page |
|---|---|---|
| Appendix 1 | Testing for a difference in risk factor effect size at a specific quantile vs. the risk factor effect size at quantile 0.5 | 2 |
| Appendix 2 | Imputation of missing data for sensitivity analysis. | 4 |
| Table S1 | Risk factor effect size estimates for ALSPAC participants at the Year 15 research clinic. | 5 |
| Table S2 | Risk factor effect size estimates for Generation R at the Year 9 research clinic. | 6 |
| Table S3 | Effect sizes quantifying associations between risk factors and refractive error, evaluated using ordinary least squares (OLS) linear regression or quantile regression after imputation of missing data using MICE. | 7 |
| Table S4 | Tests for a linear trend of increasing effect size with age. | 8 |
| Figure S1 | Comparison of effects associated with risk factor exposure estimated with ordinary least squares (OLS) linear regression or with quantile regression after imputing missing data via MICE. | 9 |
| Figure S2 | Effects associated with risk factor exposure estimated with quantile regression after imputing missing data via MICE. | 10 |

**Appendix 1. Testing for a difference in risk factor effect size at a specific quantile vs. the risk factor effect size at quantile 0.5**

The following steps were performed in R to test for a difference in risk factor effect size at a specific quantile vs. the risk factor effect size at quantile 0.5. The analysis assumes that the ratio of those exposed vs. non-exposed to the risk factor of interest is approximately 1:1.

1. Load sample dataset containing information on refractive error (coded as a continuous variable) and the risk factor of interest (coded, 1=exposed, 0=non-exposed) for $N$ participants.
2. Run a quantile regression analysis at quantiles 0.05, 0.10, 0.15, ..., 0.95 with refractive error as the dependent variable and risk factor exposure as the predictor variable.
3. Name the resulting effect size estimate at each quantile as $Eff_q$ for $q$=0.05, 0.10, 0.15, ..., 0.95 and the corresponding standard errors as $SE_q$.
4. Store the results.
5. Randomly assign each participant as being exposed or non-exposed to a simulated risk factor, i.e. create a random binomial variable (0,1).
6. Create a simulated refractive error phenotype by adding the observed median effect for the risk factor of interest ($Eff_{0.50}$) to the observed refractive error of the participant.
7. Run a quantile regression analysis at quantiles 0.05, 0.10, 0.15, ..., 0.95 with the simulated refractive error as the dependent variable and the simulated risk factor exposure as the predictor variable.
8. Name the resulting effect size estimate at each quantile as $SimEff_q$ for $q$=0.05, 0.10, 0.15, ..., 0.95 and the corresponding standard errors and variances as $SimSE_q$ and $SimVar_q$.
9. Calculate a Z-score for the difference between median and each quantile:

$$Z = \frac{SimEff_{0.50} - SimEff_q}{\sqrt{\frac{SimVar_{0.50}}{N} + \frac{SimVar_q}{N}}}$$

10. Store the resulting Z-scores.
11. Repeat steps [5-10] 10,000 times.
12. Calculate how often the observed Z-scores from step [4] would occur in the simulated data from step [11].

## R code for conducting the above analyses

```
library(quantreg)
mydata              <- [read in data file]
names(mydata)       <- c("obsMSE","obsExposed")
n                   <- dim(mydata)[1]
nperm               <- 10000

get_zscore          <- function(x1,x2,sd1,sd2,n){
a                   <- as.numeric(x1) - as.numeric(x2)
b                   <- ((as.numeric(sd1)^2)/as.numeric(n)) +
                       ((as.numeric(sd2)^2)/as.numeric(n))
c                   <- a/(b^0.5)
return(c)
}

# Quantile regression analysis for observed risk factor
# -------------------------------------------------------
results             <- as.data.frame(matrix(ncol=19,nrow=6))
row.names(results) <- c("beta","se","p","sd","Z","empircal_pval_vs_median")

# Quantile regression analysis for observed risk factor: median effect
mod_qr          <- rq(formula=obsMSE ~ obsExposed, data=mydata, tau=0.5)
mod_sum         <- summary(mod_qr)
results[1,10]   <- mod_sum$coefficients[2,1]
results[2,10]   <- mod_sum$coefficients[2,2]
results[3,10]   <- mod_sum$coefficients[2,4]
results[4,10]   <- mod_sum$coefficients[2,2] * (n^0.5)
x2              <- mod_sum$coefficients[2,1]
sd2             <- mod_sum$coefficients[2,2] * (n^0.5)

# Quantile regression analysis for observed risk factor: effect at other quantiles
for (i in 1:19){
names(results)[i]    <- paste("Q",5*i,sep="")
q               <- i/20
mod_qr          <- rq(formula=obsMSE ~ obsExposed, data=mydata, tau=q)
mod_sum         <- summary(mod_qr)
results[1,i]    <- mod_sum$coefficients[2,1]
results[2,i]    <- mod_sum$coefficients[2,2]
results[3,i]    <- mod_sum$coefficients[2,4]
results[4,i]    <- mod_sum$coefficients[2,2] * (n^0.5)
x1              <- mod_sum$coefficients[2,1]
sd1             <- mod_sum$coefficients[2,2] * (n^0.5)
if(i!=10){ results[5,i] <- abs(get_zscore(x1,x2,sd1,sd2,n)) }
}
results

# Quantile regression analysis for simulated risk factor
# --------------------------------------------------------
presults         <- as.data.frame(matrix(nrow=nperm,ncol=19))
median_effect    <- as.numeric(results[1,10])
for (p in 1:nperm){
mygroup          <- rbinom(n,size=1,prob=0.5)
myphen           <- mydata$obsMSE + (mygroup*median_effect)
mod_qrmed        <- rq(formula=myphen ~ mygroup, tau=0.5)
mod_summed       <- summary(mod_qrmed)
x2               <- mod_summed$coefficients[2,1]
sd2              <- mod_summed$coefficients[2,2]* (n^0.5)
for (i in 1:19){
q                <- i/20
mod_qr           <- rq(formula=myphen ~ mygroup, tau=q)
mod_sum          <- summary(mod_qr)
x1               <- mod_sum$coefficients[2,1]
sd1              <- mod_sum$coefficients[2,2]* (n^0.5)
if(i!=10){ presults[p,i] <- abs(get_zscore(x1,x2,sd1,sd2,n)) }c
}
}

# Calculate empirical p-values
for (i in 1:19){
if(i!=10){
 obs             <- results[5,i]
 y               <- presults[,i]
 e               <- ecdf(y)
 results[6,i]    <- 1 - e(obs)
 }
}
results
```

**Appendix 2. Imputation of missing data for sensitivity analysis.**

Missing data were imputed using Multiple Imputation by Chained Equations (MICE) [1,2] under the assumption that data were missing at random. The following variables were used for ALSPAC: Birth weight; month of birth, maternal age, maternal social class, paternal social class, gender, time outdoors, time reading, number of myopic parents, genetic risk score, number of clinic visits attended, age at Year 7 clinic visit, age at Year 10 clinic visit, age at Year 11 clinic visit, age at Year 12 clinic visit, age at Year 15 clinic visit, refractive error at Year 7 clinic visit, refractive error at Year 10 clinic visit, refractive error at Year 11 clinic visit, refractive error at Year 12 clinic visit, refractive error at Year 15 clinic visit. Similar variables were used for Generation R, only maternal and paternal social class was replaced by maternal and paternal education and net household income. Imputation was performed with the *mice* package in R, with settings of 5 imputed datasets and a maximum of 50 iterations.

The OLS linear regression and quantile regression analysis steps were repeated for the imputed data. Parameters estimates, standard errors and p-values for the quantile regression analysis pooled across imputed datasets were done under the simplifying assumption of multivariate normality.

**Table S1. Risk factor effect size estimates for ALSPAC participants at the Year 15 research clinic.** Beta coefficients correspond to the estimated effect in diopters associated with high-risk vs. low-risk status.

| Risk factor | High genetic risk score | | | At least 1 parent with myopia | | | Low time spent outdoors | | | High time spent reading | | |
|---|---|---|---|---|---|---|---|---|---|---|---|---|
| | Beta (95% C.I.) | *P*-value[1] | *P*-value[2] | Beta (95% C.I.) | *P*-value[1] | *P*-value[2] | Beta (95% C.I.) | *P*-value[1] | *P*-value[2] | Beta (95% C.I.) | *P*-value[1] | *P*-value[2] |
| <u>Quantile</u> | | | | | | | | | | | | |
| 0.05 | -1.125 (-1.479 to -0.771) | 5.01E-10 | <0.001 | -1.188 (-1.745 to -0.630) | 3.10E-05 | 0.001 | -0.750 (-1.143 to -0.357) | 1.85E-04 | 0.001 | -1.125 (-1.642 to -0.608) | 2.03E-05 | <0.001 |
| 0.10 | -0.750 (-0.990 to -0.510) | 9.49E-10 | <0.001 | -0.750 (-1.031 to -0.469) | 1.92E-07 | <0.001 | -0.375 (-0.614 to -0.136) | 2.14E-03 | 0.010 | -0.688 (-1.036 to -0.339) | 1.14E-04 | 0.001 |
| 0.15 | -0.438 (-0.590 to -0.285) | 1.83E-08 | <0.001 | -0.438 (-0.624 to -0.251) | 4.42E-06 | <0.001 | -0.188 (-0.335 to -0.040) | 1.29E-02 | 0.090 | -0.438 (-0.644 to -0.231) | 3.42E-05 | <0.001 |
| 0.20 | -0.313 (-0.416 to -0.209) | 3.61E-09 | 0.002 | -0.313 (-0.420 to -0.205) | 1.20E-08 | 0.001 | -0.125 (-0.237 to -0.013) | 2.88E-02 | 0.511 | -0.25 (-0.388 to -0.112) | 3.72E-04 | 0.002 |
| 0.25 | -0.188 (-0.258 to -0.117) | 2.34E-07 | 0.329 | -0.250 (-0.337 to -0.163) | 2.00E-08 | 0.006 | -0.063 (-0.145 to 0.020) | 1.37E-01 | 0.393 | -0.125 (-0.216 to -0.034) | 7.23E-03 | 0.374 |
| 0.30 | -0.188 (-0.243 to -0.132) | 5.75E-11 | 0.175 | -0.188 (-0.270 to -0.105) | 9.69E-06 | 0.205 | -0.063 (-0.129 to 0.004) | 6.67E-02 | 0.385 | -0.125 (-0.203 to -0.047) | 1.76E-03 | 0.339 |
| 0.35 | -0.188 (-0.243 to -0.132) | 4.07E-11 | 0.370 | -0.250 (-0.317 to -0.183) | 2.89E-13 | 0.001 | 0.000 (-0.065 to 0.065) | 1.00E+00 | 0.308 | -0.125 (-0.192 to -0.058) | 2.60E-04 | 0.298 |
| 0.40 | -0.125 (-0.184 to -0.066) | 3.49E-05 | 0.476 | -0.125 (-0.189 to -0.061) | 1.21E-04 | 0.113 | -0.063 (-0.107 to -0.018) | 6.11E-03 | 0.391 | -0.125 (-0.189 to -0.061) | 1.28E-04 | 0.304 |
| 0.45 | -0.188 (-0.236 to -0.139) | 5.20E-14 | 0.139 | -0.125 (-0.187 to -0.063) | 7.37E-05 | 0.058 | -0.063 (-0.112 to -0.013) | 1.43E-02 | 0.452 | -0.063 (-0.114 to -0.011) | 1.64E-02 | 0.454 |
| 0.50 | -0.125 (-0.173 to -0.077) | 3.73E-07 | NA | -0.125 (-0.186 to -0.064) | 6.18E-05 | NA | -0.063 (-0.105 to -0.020) | 4.28E-03 | NA | -0.063 (-0.104 to -0.021) | 2.86E-03 | NA |
| 0.55 | -0.125 (-0.174 to -0.076) | 4.93E-07 | 0.355 | -0.125 (-0.179 to -0.071) | 6.11E-06 | 0.074 | -0.063 (-0.096 to -0.029) | 2.41E-04 | 0.511 | -0.063 (-0.097 to -0.028) | 3.21E-04 | 0.498 |
| 0.60 | -0.125 (-0.175 to -0.075) | 1.07E-06 | 0.420 | -0.188 (-0.243 to -0.132) | 5.28E-11 | 0.078 | -0.063 (-0.114 to -0.011) | 1.75E-02 | 0.538 | -0.063 (-0.115 to -0.010) | 2.00E-02 | 0.540 |
| 0.65 | -0.188 (-0.240 to -0.135) | 3.32E-12 | 0.159 | -0.125 (-0.183 to -0.067) | 2.88E-05 | 0.139 | -0.063 (-0.108 to -0.017) | 6.55E-03 | 0.324 | -0.063 (-0.118 to -0.007) | 2.65E-02 | 0.301 |
| 0.70 | -0.063 (-0.110 to -0.015) | 1.01E-02 | 0.555 | -0.125 (-0.187 to -0.063) | 8.35E-05 | 0.168 | -0.063 (-0.110 to -0.015) | 1.06E-02 | 0.578 | -0.063 (-0.114 to -0.011) | 1.84E-02 | 0.574 |
| 0.75 | -0.125 (-0.176 to -0.074) | 1.69E-06 | 0.835 | -0.125 (-0.206 to -0.044) | 2.58E-03 | 0.233 | 0.000 (-0.062 to 0.062) | 1.00E+00 | 0.797 | 0.000 (-0.051 to 0.051) | 1.00E+00 | 0.097 |
| 0.80 | -0.188 (-0.243 to -0.132) | 4.51E-11 | 0.242 | -0.188 (-0.269 to -0.106) | 6.90E-06 | 0.194 | -0.063 (-0.130 to 0.005) | 6.85E-02 | 0.568 | -0.063 (-0.131 to 0.006) | 7.44E-02 | 0.545 |
| 0.85 | -0.188 (-0.260 to -0.115) | 3.56E-07 | 0.490 | -0.188 (-0.285 to -0.090) | 1.61E-04 | 0.224 | -0.063 (-0.137 to 0.012) | 9.82E-02 | 0.649 | -0.063 (-0.129 to 0.004) | 6.72E-02 | 0.626 |
| 0.90 | -0.188 (-0.310 to -0.065) | 2.70E-03 | 0.652 | -0.313 (-0.486 to -0.139) | 4.26E-04 | 0.015 | -0.063 (-0.207 to 0.082) | 3.96E-01 | 0.774 | -0.063 (-0.188 to 0.063) | 3.28E-01 | 0.730 |
| 0.95 | -0.188 (-0.564 to 0.189) | 3.29E-01 | 0.801 | -0.938 (-1.628 to -0.247) | 7.80E-03 | 0.003 | 0.000 (-0.410 to 0.410) | 1.00E+00 | 0.720 | 0.063 (-0.361 to 0.486) | 7.73E-01 | 0.537 |

[1] *P*-value for test of null hypothesis that beta coefficient at that quantile equals zero.

[2] *P*-value for test of null hypothesis that the beta coefficient at that quantile is the same magnitude as the beta coefficient for quantile 0.50.

**Table S2. Risk factor effect size estimates for Generation R at the Year 9 research clinic.** Beta coefficients correspond to the estimated effect in diopters for the high-risk vs. low-risk group.

| Risk factor | High genetic risk score | | | At least 1 parent with myopia | | | Low time spent outdoors | | | High time spent reading | | |
|---|---|---|---|---|---|---|---|---|---|---|---|---|
| | Beta (95% C.I.) | *P*-value[1] | *P*-value[2] | Beta (95% C.I.) | *P*-value[1] | *P*-value[2] | Beta (95% C.I.) | *P*-value[1] | *P*-value[2] | Beta (95% C.I.) | *P*-value[1] | *P*-value[2] |
| **Quantile** | | | | | | | | | | | | |
| 0.05 | -1.063 (-1.685 to -0.440) | 8.43E-04 | 0.007 | -1.313 (-1.801 to -0.824) | 1.60E-07 | <0.001 | -0.313 (-0.850 to 0.225) | 2.54E-01 | 0.160 | -0.438 (-1.117 to 0.242) | 2.07E-01 | 0.358 |
| 0.10 | -0.750 (-1.119 to -0.381) | 7.10E-05 | 0.003 | -0.875 (-1.168 to -0.582) | 5.97E-09 | <0.001 | -0.250 (-0.573 to 0.073) | 1.29E-01 | 0.041 | -0.375 (-0.792 to 0.042) | 7.79E-02 | 0.208 |
| 0.15 | -0.563 (-0.784 to -0.341) | 6.92E-07 | 0.002 | -0.438 (-0.674 to -0.201) | 3.05E-04 | 0.013 | -0.063 (-0.279 to 0.154) | 5.72E-01 | 0.211 | -0.250 (-0.494 to -0.006) | 4.47E-02 | 0.075 |
| 0.20 | -0.50 (-0.678 to -0.322) | 4.36E-08 | 0.002 | -0.313 (-0.474 to -0.151) | 1.56E-04 | 0.089 | 0.063 (-0.081 to 0.206) | 3.93E-01 | 0.664 | -0.125 (-0.298 to 0.048) | 1.56E-01 | 0.454 |
| 0.25 | -0.438 (-0.595 to -0.280) | 6.09E-08 | 0.016 | -0.250 (-0.384 to -0.116) | 2.58E-04 | 0.537 | -0.063 (-0.178 to 0.053) | 2.88E-01 | 0.006 | -0.063 (-0.203 to 0.078) | 3.83E-01 | 0.650 |
| 0.30 | -0.313 (-0.434 to -0.191) | 5.08E-07 | 0.152 | -0.250 (-0.350 to -0.150) | 1.09E-06 | 0.132 | 0.000 (-0.085 to 0.085) | 1.00E+00 | 0.016 | -0.063 (-0.176 to 0.051) | 2.82E-01 | 0.556 |
| 0.35 | -0.313 (-0.428 to -0.197) | 1.27E-07 | 0.338 | -0.188 (-0.286 to -0.089) | 2.09E-04 | 0.472 | 0.000 (-0.088 to 0.088) | 1.00E+00 | 0.110 | 0.000 (-0.092 to 0.092) | 1.00E+00 | 0.271 |
| 0.40 | -0.250 (-0.343 to -0.157) | 1.77E-07 | 0.754 | -0.188 (-0.269 to -0.106) | 7.79E-06 | 0.879 | 0.000 (-0.055 to 0.055) | 1.00E+00 | <0.001 | -0.063 (-0.129 to 0.004) | 6.67E-02 | 0.815 |
| 0.45 | -0.250 (-0.335 to -0.165) | 9.31E-09 | 0.294 | -0.188 (-0.263 to -0.112) | 1.32E-06 | 0.521 | 0.063 (0.000 to 0.125) | 4.93E-02 | 0.200 | -0.063 (-0.138 to 0.013) | 1.06E-01 | 0.525 |
| 0.50 | -0.250 (-0.325 to -0.175) | 9.68E-11 | NA | -0.188 (-0.262 to -0.113) | 1.02E-06 | NA | 0.063 (0.003 to 0.122) | 4.06E-02 | NA | -0.063 (-0.142 to 0.017) | 1.26E-01 | NA |
| 0.55 | -0.250 (-0.335 to -0.165) | 9.31E-09 | 0.413 | -0.188 (-0.267 to -0.108) | 4.04E-06 | 0.898 | 0.000 (-0.058 to 0.058) | 1.00E+00 | 0.001 | -0.063 (-0.136 to 0.011) | 9.67E-02 | 0.698 |
| 0.60 | -0.125 (-0.209 to -0.041) | 3.49E-03 | <0.001 | -0.125 (-0.190 to -0.060) | 1.57E-04 | 0.134 | 0.000 (-0.062 to 0.062) | 1.00E+00 | 0.032 | -0.063 (-0.134 to 0.009) | 8.82E-02 | 0.776 |
| 0.65 | -0.188 (-0.275 to -0.100) | 3.04E-05 | 0.177 | -0.125 (-0.195 to -0.055) | 4.49E-04 | 0.008 | 0.000 (-0.063 to 0.063) | 1.00E+00 | 0.041 | -0.063 (-0.140 to 0.015) | 1.16E-01 | 0.864 |
| 0.70 | -0.250 (-0.337 to -0.163) | 2.44E-08 | 0.353 | -0.188 (-0.268 to -0.107) | 4.93E-06 | 0.411 | 0.000 (-0.083 to 0.083) | 1.00E+00 | 0.213 | -0.063 (-0.155 to 0.030) | 1.88E-01 | 0.469 |
| 0.75 | -0.250 (-0.346 to -0.154) | 3.86E-07 | 0.667 | -0.125 (-0.214 to -0.036) | 6.11E-03 | 0.414 | -0.063 (-0.134 to 0.009) | 8.72E-02 | 0.003 | 0.000 (-0.078 to 0.078) | 1.00E+00 | 0.398 |
| 0.80 | -0.250 (-0.364 to -0.136) | 1.87E-05 | 0.455 | -0.125 (-0.236 to -0.014) | 2.68E-02 | 0.453 | 0.000 (-0.091 to 0.091) | 1.00E+00 | 0.174 | -0.063 (-0.159 to 0.034) | 2.06E-01 | 0.509 |
| 0.85 | -0.250 (-0.390 to -0.110) | 4.90E-04 | 0.733 | -0.188 (-0.356 to -0.019) | 2.94E-02 | 0.767 | -0.063 (-0.199 to 0.074) | 3.69E-01 | 0.076 | -0.125 (-0.252 to 0.002) | 5.32E-02 | 0.158 |
| 0.90 | -0.563 (-0.922 to -0.203) | 2.19E-03 | 0.074 | -0.438 (-0.720 to -0.155) | 2.45E-03 | 0.052 | -0.125 (-0.372 to 0.122) | 3.22E-01 | 0.110 | -0.188 (-0.482 to 0.107) | 2.12E-01 | 0.300 |
| 0.95 | -0.750 (-1.155 to -0.345) | 2.90E-04 | 0.008 | -0.375 (-0.860 to 0.110) | 1.30E-01 | 0.466 | -0.188 (-0.578 to 0.203) | 3.47E-01 | 0.141 | -0.313 (-0.740 to 0.115) | 1.52E-01 | 0.138 |

[1] *P*-value for test of null hypothesis that beta coefficient at that quantile equals zero.

[2] *P*-value for test of null hypothesis that the beta coefficient at that quantile is the same magnitude as the beta coefficient for quantile 0.50.

**Table S3. Effect sizes quantifying associations between risk factors and refractive error, evaluated using ordinary least squares (OLS) linear regression or quantile regression after imputation of missing data using MICE.**

| Risk factor | Cohort | Research clinic | N | Ordinary Least Squares regression | | Quantile regression: quantile 0.05 | | Quantile regression: quantile 0.50 | |
|---|---|---|---|---|---|---|---|---|---|
| | | | | Beta (95% CI) | *P*-value | Beta (95% CI) | *P*-value | Beta (95% CI) | *P*-value |
| Female gender | ALSPAC | Year 7 | 6504 | -0.009 (-0.054 to 0.035) | 6.83E-01 | 0.000 (-0.077 to 0.077) | 1.00E+00 | 0.000 (-0.029 to 0.029) | 1.00E+00 |
| | | Year 10 | 6504 | 0.001 (-0.054 to 0.056) | 9.69E-01 | 0.012 (-0.168 to 0.193) | 8.92E-01 | 0.000 (-0.037 to 0.037) | 1.00E+00 |
| | | Year 12 | 6504 | -0.046 (-0.107 to 0.015) | 1.38E-01 | -0.138 (-0.408 to 0.133) | 3.20E-01 | 0.000 (-0.029 to 0.029) | 1.00E+00 |
| | | Year 15 | 6504 | -0.027 (-0.104 to 0.049) | 4.92E-01 | -0.050 (-0.400 to 0.300) | 7.80E-01 | -0.025 (-0.106 to 0.056) | 5.68E-01 |
| | Generation R | Year 9 | 2395 | 0.01 (-0.091 to 0.111) | 8.47E-01 | -0.062 (-0.585 to 0.46) | 8.14E-01 | 0.062 (-0.003 to 0.128) | 6.20E-02 |
| High genetic risk | ALSPAC | Year 7 | 6504 | -0.165 (-0.211 to -0.119) | 9.30E-12 | -0.188 (-0.266 to -0.109) | 2.60E-06 | -0.125 (-0.152 to -0.098) | <2.00E-16 |
| | | Year 10 | 6504 | -0.242 (-0.298 to -0.185) | 6.66E-16 | -0.488 (-0.673 to -0.302) | 4.34E-07 | -0.138 (-0.204 to -0.071) | 6.55E-03 |
| | | Year 12 | 6504 | -0.290 (-0.351 to -0.228) | <2.00E-16 | -0.875 (-1.129 to -0.621) | 1.45E-10 | -0.125 (-0.154 to -0.096) | <2.00E-16 |
| | | Year 15 | 6504 | -0.344 (-0.413 to -0.275) | <2.00E-16 | -1.075 (-1.362 to -0.788) | 8.20E-12 | -0.125 (-0.155 to -0.095) | 2.22E-16 |
| | Generation R | Year 9 | 2395 | -0.278 (-0.389 to -0.166) | 2.98E-06 | -0.5 (-1.158 to 0.158) | 1.53E-01 | -0.138 (-0.226 to -0.049) | 6.59E-03 |
| Has myopic parent(s) | ALSPAC | Year 7 | 6504 | -0.244 (-0.313 to -0.175) | 2.00E-05 | -0.212 (-0.353 to -0.072) | 1.94E-02 | -0.125 (-0.155 to -0.095) | <2.00E-16 |
| | | Year 10 | 6504 | -0.335 (-0.404 to -0.266) | 3.39E-10 | -0.675 (-0.843 to -0.507) | 1.10E-13 | -0.112 (-0.229 to 0.004) | 1.22E-01 |
| | | Year 12 | 6504 | -0.398 (-0.488 to -0.308) | 1.27E-06 | -1.062 (-1.377 to -0.748) | 2.42E-07 | -0.125 (-0.155 to -0.095) | <2.00E-16 |
| | | Year 15 | 6504 | -0.450 (-0.553 to -0.347) | 5.17E-06 | -1.325 (-1.641 to -1.009) | 2.51E-10 | -0.175 (-0.242 to -0.108) | 1.99E-03 |
| | Generation R | Year 9 | 2395 | -0.326 (-0.446 to -0.207) | 2.38E-06 | -0.938 (-1.418 to -0.457) | 1.60E-04 | -0.175 (-0.257 to -0.093) | 9.28E-04 |
| Time spent reading high | ALSPAC | Year 7 | 6504 | -0.055 (-0.103 to -0.007) | 2.56E-02 | -0.150 (-0.301 to 0.001) | 8.03E-02 | 0.000 (-0.030 to 0.030) | 1.00E+00 |
| | | Year 10 | 6504 | -0.116 (-0.173 to -0.059) | 6.71E-05 | -0.462 (-0.711 to -0.214) | 4.84E-04 | 0.000 (-0.032 to 0.032) | 1.00E+00 |
| | | Year 12 | 6504 | -0.167 (-0.236 to -0.098) | 7.61E-06 | -0.750 (-1.118 to -0.382) | 3.05E-04 | -0.038 (-0.117 to 0.042) | 3.94E-01 |
| | | Year 15 | 6504 | -0.203 (-0.272 to -0.134) | 1.43E-08 | -0.912 (-1.281 to -0.544) | 1.64E-05 | -0.062 (-0.099 to -0.026) | 9.26E-04 |
| | Generation R | Year 9 | 2395 | -0.285 (-0.529 to -0.04) | 3.07E-02 | -0.862 (-1.407 to -0.318) | 1.49E-03 | -0.162 (-0.292 to -0.033) | 1.83E-02 |
| Time spent outdoors low | ALSPAC | Year 7 | 6504 | -0.062 (-0.114 to -0.009) | 2.54E-02 | -0.112 (-0.202 to -0.023) | 2.30E-02 | 0.000 (-0.029 to 0.029) | 1.00E+00 |
| | | Year 10 | 6504 | -0.093 (-0.158 to -0.027) | 8.22E-03 | -0.325 (-0.607 to -0.043) | 4.57E-02 | -0.012 (-0.079 to 0.054) | 7.26E-01 |
| | | Year 12 | 6504 | -0.130 (-0.198 to -0.062) | 3.49E-04 | -0.512 (-0.864 to -0.161) | 1.03E-02 | 0.000 (-0.029 to 0.029) | 1.00E+00 |
| | | Year 15 | 6504 | -0.145 (-0.215 to -0.074) | 9.83E-05 | -0.750 (-1.065 to -0.435) | 7.86E-06 | -0.075 (-0.144 to -0.006) | 7.08E-02 |
| | Generation R | Year 9 | 2395 | -0.055 (-0.168 to 0.057) | 3.38E-01 | -0.238 (-0.749 to 0.274) | 3.63E-01 | 0.025 (-0.070 to 0.120) | 6.17E-01 |

**Table S4. Tests for a linear trend of increasing effect size with age.** A negative beta coefficient indicates an increasingly negative (more myopic) effect size with age.

| Quantile | Female gender | | High genetic risk | | Has myopic parent(s) | | Time spent reading high | | Time spent outdoors low | |
|---|---|---|---|---|---|---|---|---|---|---|
| | Beta (95% CI) | *P*-value | Beta (95% CI) | *P*-value | Beta (95% CI) | *P*-value | Beta (95% CI) | *P*-value | Beta (95% CI) | *P*-value |
| 0.05 | -0.007 (-0.048 to 0.034) | 7.32e-01 | -0.114 (-0.149 to -0.079) | 1.95e-10 | -0.136 (-0.183 to -0.088) | 2.58e-08 | -0.109 (-0.158 to -0.059) | 1.59e-05 | -0.09 (-0.126 to -0.054) | 9.20e-07 |
| 0.10 | -0.011 (-0.032 to 0.009) | 2.81e-01 | -0.069 (-0.096 to -0.041) | 8.08e-07 | -0.076 (-0.101 to -0.051) | 1.61e-09 | -0.073 (-0.101 to -0.045) | 3.94e-07 | -0.055 (-0.078 to -0.031) | 4.31e-06 |
| 0.15 | -0.020 (-0.039 to -0.001) | 3.99e-02 | -0.029 (-0.052 to -0.005) | 1.59e-02 | -0.041 (-0.063 to -0.019) | 1.98e-04 | -0.034 (-0.052 to -0.015) | 4.32e-04 | -0.018 (-0.037 to 0.001) | 6.73e-02 |
| 0.20 | -0.010 (-0.021 to 0.000) | 5.15e-02 | -0.024 (-0.035 to -0.013) | 2.74e-05 | -0.024 (-0.036 to -0.011) | 2.09e-04 | -0.031 (-0.045 to -0.017) | 1.93e-05 | -0.020 (-0.032 to -0.008) | 7.43e-04 |
| 0.25 | -0.009 (-0.019 to 0.001) | 8.17e-02 | -0.012 (-0.031 to 0.008) | 2.37e-01 | -0.015 (-0.026 to -0.005) | 5.16e-03 | -0.019 (-0.029 to -0.008) | 6.23e-04 | -0.012 (-0.031 to 0.007) | 2.22e-01 |
| 0.30 | -0.010 (-0.020 to 0.000) | 4.74e-02 | -0.010 (-0.019 to -0.001) | 3.80e-02 | -0.010 (-0.020 to 0.000) | 4.32e-02 | -0.014 (-0.023 to -0.005) | 2.75e-03 | -0.009 (-0.017 to -0.002) | 1.81e-02 |
| 0.35 | 0.000 (-0.007 to 0.007) | 1.00e+00 | -0.005 (-0.013 to 0.002) | 1.44e-01 | -0.012 (-0.029 to 0.005) | 1.68e-01 | -0.015 (-0.023 to -0.006) | 5.02e-04 | -0.002 (-0.016 to 0.013) | 8.42e-01 |
| 0.40 | -0.006 (-0.013 to 0.001) | 1.05e-01 | 0.000 (-0.007 to 0.007) | 1.00e+00 | 0.000 (-0.008 to 0.008) | 1.00e+00 | -0.015 (-0.023 to -0.007) | 2.62e-04 | -0.007 (-0.014 to 0.001) | 8.61e-02 |
| 0.45 | -0.006 (-0.013 to 0.001) | 1.01e-01 | -0.006 (-0.013 to 0.002) | 1.37e-01 | 0.000 (-0.008 to 0.008) | 1.00e+00 | -0.006 (-0.013 to 0.001) | 1.01e-01 | -0.005 (-0.019 to 0.008) | 4.26e-01 |
| 0.50 | -0.006 (-0.014 to 0.002) | 1.48e-01 | 0.000 (-0.006 to 0.006) | 1.00e+00 | 0.000 (-0.008 to 0.008) | 1.00e+00 | -0.009 (-0.016 to -0.002) | 1.14e-02 | -0.007 (-0.014 to 0.001) | 8.46e-02 |
| 0.55 | 0.001 (-0.014 to 0.016) | 9.25e-01 | -0.008 (-0.017 to 0.000) | 5.00e-02 | 0.000 (-0.007 to 0.007) | 1.00e+00 | -0.002 (-0.015 to 0.011) | 7.89e-01 | 0.000 (-0.015 to 0.015) | 9.77e-01 |
| 0.60 | 0.008 (0.000 to 0.017) | 4.90e-02 | -0.008 (-0.017 to 0.000) | 4.91e-02 | 0.003 (-0.011 to 0.016) | 7.13e-01 | -0.001 (-0.015 to 0.012) | 8.37e-01 | 0.001 (-0.014 to 0.016) | 9.16e-01 |
| 0.65 | 0.005 (-0.008 to 0.019) | 4.53e-01 | -0.014 (-0.021 to -0.007) | 5.43e-05 | 0.008 (-0.001 to 0.018) | 7.97e-02 | -0.01 (-0.019 to 0.000) | 4.50e-02 | -0.006 (-0.015 to 0.002) | 1.20e-01 |
| 0.70 | 0.007 (0.000 to 0.015) | 6.46e-02 | -0.001 (-0.015 to 0.014) | 9.46e-01 | 0.010 (-0.006 to 0.026) | 2.23e-01 | -0.002 (-0.016 to 0.012) | 7.81e-01 | 0.002 (-0.011 to 0.015) | 7.35e-01 |
| 0.75 | 0.000 (-0.007 to 0.007) | 1.00e+00 | 0.000 (-0.007 to 0.007) | 1.00e+00 | 0.009 (-0.002 to 0.020) | 1.26e-01 | 0.005 (-0.008 to 0.019) | 4.32e-01 | 0.002 (-0.012 to 0.016) | 7.94e-01 |
| 0.80 | 0.006 (-0.003 to 0.015) | 2.07e-01 | -0.007 (-0.015 to 0.002) | 1.21e-01 | 0.003 (-0.011 to 0.017) | 6.60e-01 | -0.005 (-0.019 to 0.009) | 4.52e-01 | -0.009 (-0.018 to 0.000) | 5.33e-02 |
| 0.85 | 0.005 (-0.008 to 0.019) | 4.55e-01 | -0.005 (-0.019 to 0.010) | 5.05e-01 | 0.009 (-0.006 to 0.025) | 2.43e-01 | 0.002 (-0.013 to 0.017) | 7.73e-01 | -0.005 (-0.019 to 0.009) | 4.66e-01 |
| 0.90 | 0.000 (-0.017 to 0.016) | 9.85e-01 | 0.010 (-0.008 to 0.027) | 2.84e-01 | 0.007 (-0.02 to 0.034) | 6.14e-01 | 0.007 (-0.027 to 0.041) | 6.80e-01 | 0.009 (-0.01 to 0.028) | 3.59e-01 |
| 0.95 | -0.003 (-0.049 to 0.043) | 8.97e-01 | 0.014 (-0.041 to 0.069) | 6.23e-01 | -0.057 (-0.155 to 0.04) | 2.50e-01 | 0.011 (-0.045 to 0.066) | 7.03e-01 | 0.024 (-0.030 to 0.077) | 3.88e-01 |
| **OLS** | 0.000 (-0.015 to 0.014) | 9.53E-01 | -0.024 (-0.035 to -0.013) | 8.42E-06 | -0.023 (-0.036 to -0.010) | 5.35E-04 | -0.018 (-0.030 to -0.006) | 2.48E-03 | -0.011 (-0.022 to 0.001) | 6.81E-02 |

**Figure S1. Comparison of effects associated with risk factor exposure estimated with ordinary least squares (OLS) linear regression or with quantile regression after imputing missing data via MICE.** Panel A, refractive error at the Year 15 research clinic in ALSPAC participants. Panel B, refractive error at the Year 9 research clinic in Generation R participants. The dashed blue line indicates the effect associated with exposure to the risk factor, calculated with OLS linear regression (95% confidence interval shown as light blue shaded region). Filled circles correspond to the effect associated with each exposure, calculated with quantile regression (error bars indicate 95% confidence interval). Note that effect sizes can vary across quantiles of the refractive error distribution for quantile regression.

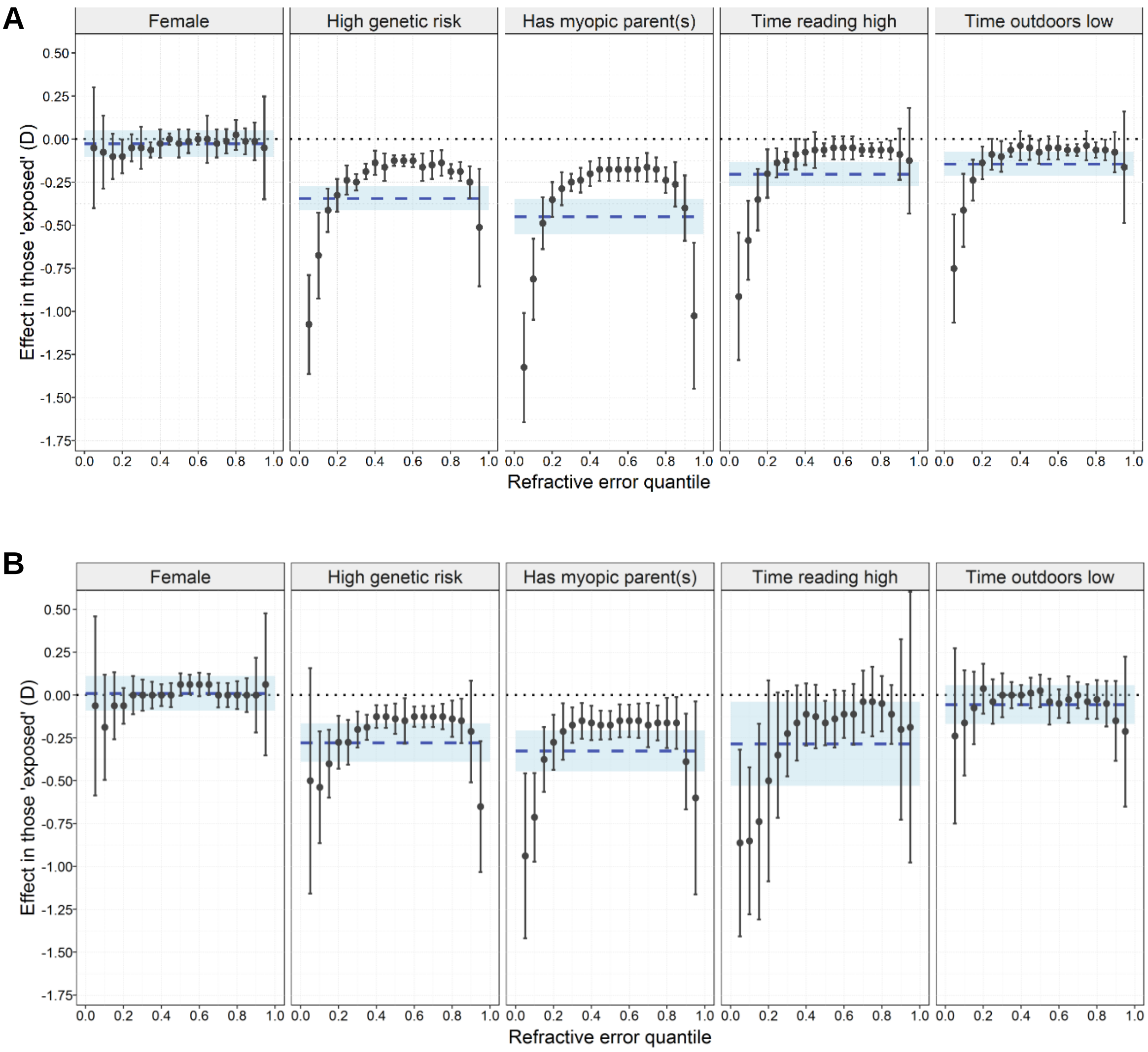

**Figure S2. Effects associated with risk factor exposure estimated with quantile regression after imputing missing data via MICE.** Panel A, refractive error at the Year 7 to Year 15 research clinics in ALSPAC participants. Panel B, refractive error at the Year 9 research clinic in Generation R participants. The fitted lines indicate the effect associated with exposure to the risk factor (shaded regions indicate 95% confidence interval of Loess fit).

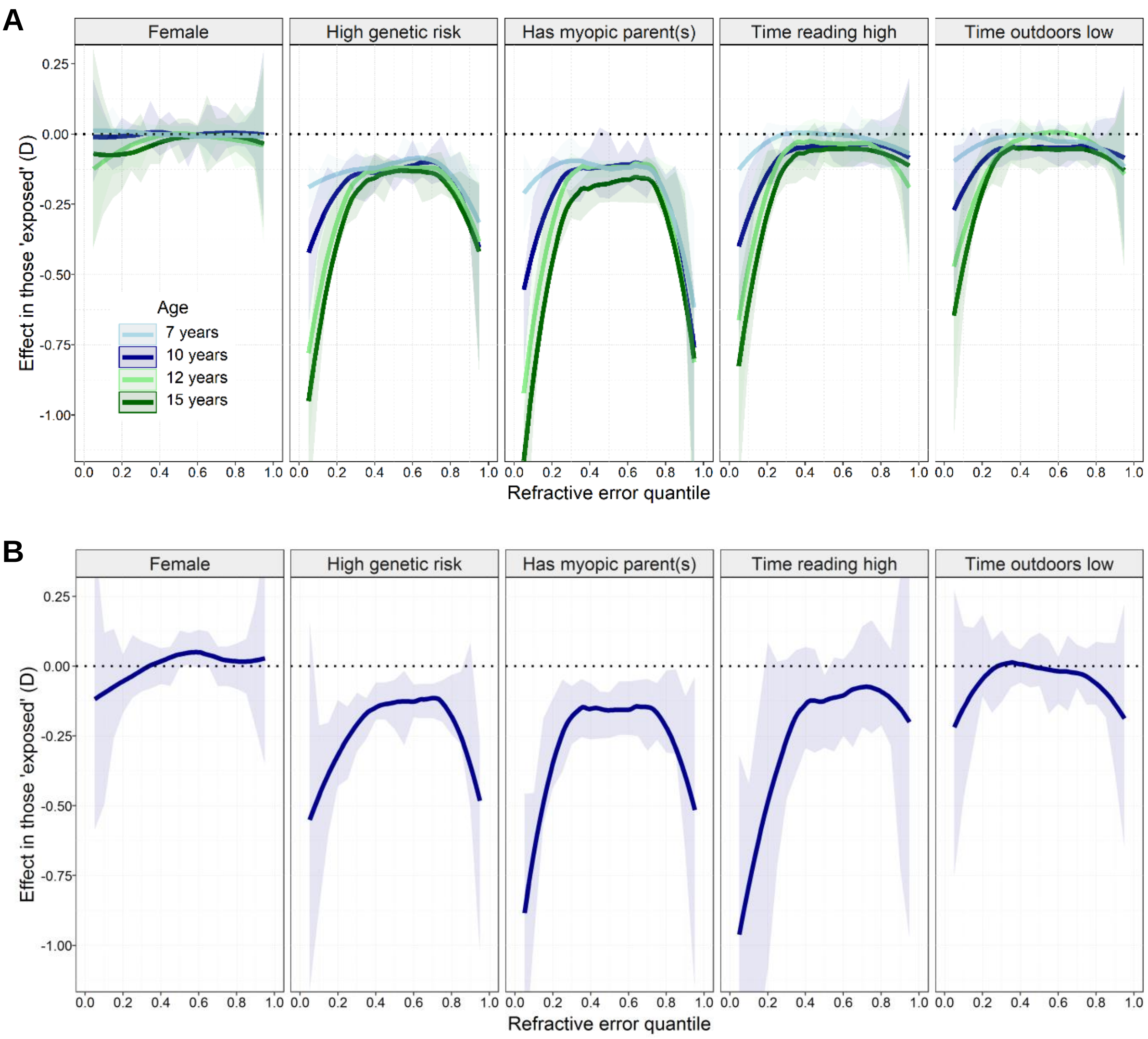

## References


1. Sterne JAC, White IR, Carlin JB, et al. Multiple imputation for missing data in epidemiological and clinical research: potential and pitfalls. *Br Med J* 2009;339:b2393.
2. Royston P. Multiple imputation of missing values. *STATA Journal* 2004;4:227-41.
